# Crowded Out

**Published:** 1884-04

**Authors:** 


### CROWDED OUT.

The inexorable fiat of the printer again shuts out considerable matter that had been prepared for this number. "Our Book Table" shares the fate of sundry editorials, and we have been obliged, greatly to our regret, to condense the announcements of coming meetings, as well as to reduce sundry other articles from a page to a paragraph.

